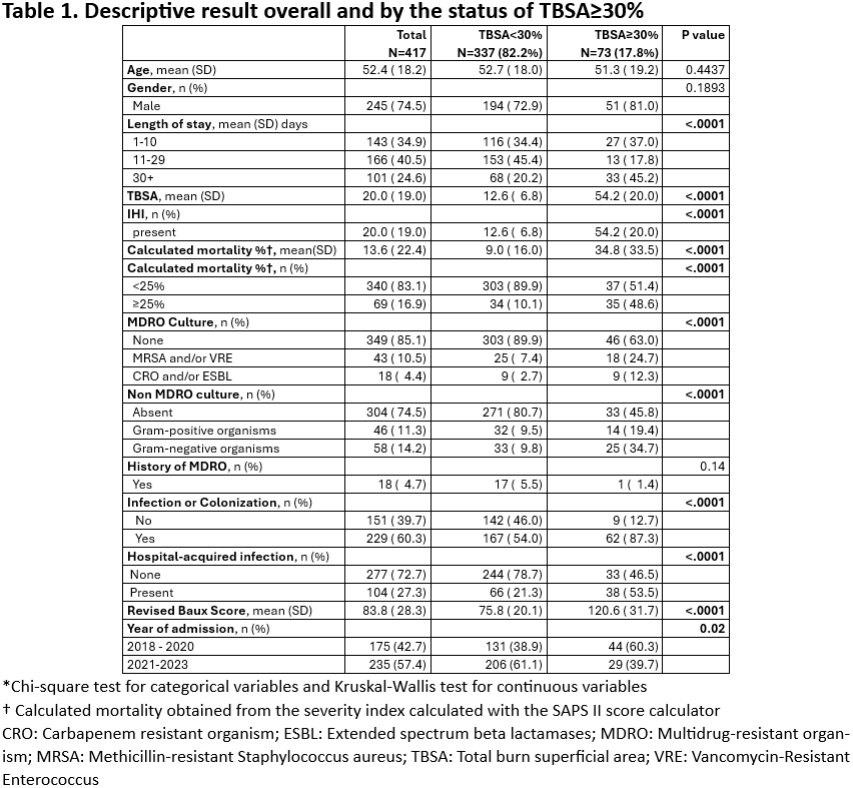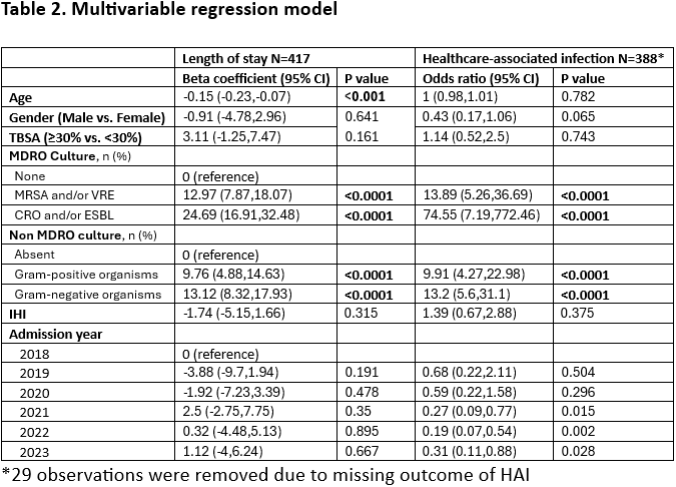# 890 Retrospective Review of Critically-ill Burn Patients and Multidrug-resistant Organism Infection and Colonization

**DOI:** 10.1093/jbcr/iraf019.421

**Published:** 2025-04-01

**Authors:** Genesis Huerta, Shehryar Malik, Jenny Ziembicki, Francesco Egro, Yassin Mohamed, Chris Musgrove, Dianxu Ren

**Affiliations:** University of Pittsburgh Medical Center, Mercy Burn Center; University of Pittsburgh Medical Center; University of Pittsburgh Medical Center; University of Pittsburgh Medical Center; University of Pittsburgh; University of Iowa; University of Pittsburgh

## Abstract

**Introduction:**

The National Burn Repository reports on major percentage of burn victims and recognizes total burn surface area (TBSA) and inhalational lung injury as major risk factors for burn outcomes. Infections are the most serious and most common complication of burns. Patients with serious burn injuries typically have long length of stay (LOS) and are vulnerable to many infections and even death. The primary aim of the study was to evaluate the presence of healthcare-associated infections (HAI), and colonization particularly multidrug resistant organisms (MDRO).

**Methods:**

Our burn center serves around 3000 patients annually and less than 10% of these patients require hospital or intensive care admission. We reviewed burn victims over the past 6 years (January 1st, 2018 till Dec., 31st, 2023). Inclusion criteria included adult patients with either TBSA of 10% or more and /or evidence of ILI. Patients who died within 72 hours of admission were excluded from the study. The electronic health records were reviewed to determine burn characteristics, demographics, infection, and microbiology results. Microbiology data utilized historical, clinical, and surveillance data. Additionally, the severity index was calculated on admission to inpatient burn care. Statistic associations were obtained with chi-square test and Kruskal-Wallis test. A regression model was performed for length of stay (LOS) and HAI.

**Results:**

During this 6-year review, we identified 426 patients who fulfilled the inclusion criteria. Table 1 includes baseline characteristics, including microbiologic data. Positive cultures for any bacteria were present for 25.3% of the population and 14.8% had cultures positive for MDRO. HAI was present in 27.1%: Bone and soft tissue 12.11%, Pneumonia 7.99%, UTI 4.12%, and bacteremia 2.84%. Healthcare-acquired infection and calculated mortality were associated with TBSA ≥30% (p < 0.0001) as shown in Table 1.

Any type of positive culture was associated with longer LOS and higher risk of HAI, however, MDRO cultures had higher OR, more specifically positive CRO or ESBL (Table 2).

**Conclusions:**

Our data were not different than the national repository reports. Critically ill burn victims are at high risk for healthcare-associated infection and colonization, particularly with multidrug-resistant organisms. Inhalational lung injury is a particularly important risk factor for infection and colonization. The retrospective review of the data helped understand the challenges of identifying other Gram-negative colonization for which there was no routine surveillance.

**Applicability of Research to Practice:**

Microbiologic epidemiology information from a large burn center with specific information about MDRO colonization

**Funding for the Study:**

Internal funding from the institution to cover data analysis and collection